# Effectiveness of a Video-Versus Text-Based Computer-Tailored Intervention for Obesity Prevention after One Year: A Randomized Controlled Trial

**DOI:** 10.3390/ijerph14101275

**Published:** 2017-10-23

**Authors:** Kei Long Cheung, Inga Schwabe, Michel J. L. Walthouwer, Anke Oenema, Lilian Lechner, Hein de Vries

**Affiliations:** 1 Department of Health Services Research, Care and Public Health Research Institute (CAPHRI), Maastricht University, 6211 LK Maastricht, The Netherlands; 2 Department of Health Promotion, Care and Public Health Research Institute (CAPHRI), Maastricht University, 6211 LK Maastricht, The Netherlands; m.walthouwer@maastrichtuniversity.nl (M.J.L.W.); a.oenema@maastrichtuniversity.nl (A.O.); hein.devries@maastrichtuniversity.nl (H.d.V.); 3 Department of Methodology and Statistics, School of Social and Behavioral Sciences (TSB), Tilburg University, 5037 AB Tilburg, The Netherlands; I.Schwabe@uvt.nl; 4 Department of Psychology, Open University of the Netherlands, 6419 AT Heerlen, The Netherlands; Lilian.Lechner@ou.nl

**Keywords:** randomized controlled trial, web-based, computer-tailoring, obesity, educational level, energy intake

## Abstract

Computer-tailored programs may help to prevent overweight and obesity, which are worldwide public health problems. This study investigated (1) the 12-month effectiveness of a video- and text-based computer-tailored intervention on energy intake, physical activity, and body mass index (BMI), and (2) the role of educational level in intervention effects. A randomized controlled trial in The Netherlands was conducted, in which adults were allocated to a video-based condition, text-based condition, or control condition, with baseline, 6 months, and 12 months follow-up. Outcome variables were self-reported BMI, physical activity, and energy intake. Mixed-effects modelling was used to investigate intervention effects and potential interaction effects. Compared to the control group, the video intervention group was effective regarding energy intake after 6 months (least squares means (LSM) difference = −205.40, *p* = 0.00) and 12 months (LSM difference = −128.14, *p* = 0.03). Only video intervention resulted in lower average daily energy intake after one year (d = 0.12). Educational role and BMI did not seem to interact with this effect. No intervention effects on BMI and physical activity were found. The video computer-tailored intervention was effective on energy intake after one year. This effect was not dependent on educational levels or BMI categories, suggesting that video tailoring can be effective for a broad range of risk groups and may be preferred over text tailoring.

## 1. Introduction

Worldwide, overweight and obesity are among the most important public health challenges, with obesity rates having doubled since 1980 [1]. This is reflected by the body mass index (BMI), commonly used to classify overweight and obesity in adults [2]. In 2014, more than 1.9 billion adults (i.e., 18 years and older) were overweight (i.e., BMI greater than or equal to 25), from which 600 million adults were obese (i.e., BMI greater than or equal to 30) [3]. In The Netherlands, almost half of Dutch adults classified as overweight in 2016, while 14.2% of adults classified as obese [4]. Literature shows that the prevalence of overweight and obesity is significantly higher among people with a low level of education [5,6,7]. In The Netherlands, estimates regarding overweight in Dutch adults revealed more than 20% difference between lower- and higher-educated adults in 2016 [4]. To tackle the scourge of overweight and obesity, interventions need to reach many people in an efficacious yet cost-effective manner, as overweight and obesity affect a large number of people [8]. 

In a systematic review of randomized controlled trials which evaluate the effectiveness of eHealth interventions for the prevention and treatment of overweight and obesity in adults, support was found for the use of eHealth interventions as a treatment option for obesity. Yet there is insufficient evidence regarding obesity prevention, and there is a need for more research regarding their effects [9]. A promising approach to prevent obesity is the use of computer-tailored interventions. Computer-tailored interventions are characterized by the provision of feedback (in the form of health messages) adapted to the users’ characteristics to target health-related behaviors, such as physical activity and energy intake [10]. Computer tailoring has been shown to be effective in improving health behaviors (e.g., physical activity, adjusting body weight, and eating a healthy diet) [8,11]. Web-based computer-tailored interventions have the opportunity to reach many people with low costs per person (enhancing the likelihood of cost-effectiveness) [12,13]. Moreover, a recent study in The Netherlands indicated that the usage of eHealth interventions as recommended did not differ among education and income levels [14]. This means that computer-tailored interventions also have great opportunity to support lower-educated individuals. Furthermore, the use of audio-visual aids may improve such interventions, due to a potential better fit of the delivery format and the user’s preferences [15], regardless of the educational level of its participants. 

A recent computer-tailored intervention—aimed to prevent weight gain or help adults achieve modest weight loss by improving dietary intake (hereafter: energy intake (from energy-dense food products)), physical activity, or both—with video- and text-based versions showed promising short-term effects [16]. Web-based computer-tailored interventions may have problems reaching the people with a low educational level, who often are most in need of change [17]. As lower-educated individuals typically have more difficulties processing large amounts of text [18], it has been argued that video messages may be more appealing to adults with lower educational levels due to the reduced cognitive effort needed to process information [19]. Hence, this recent computer-tailored intervention incorporated a delivery of intervention content via videos. The 6-month follow-up study examined intervention effects and the potential differences per educational level. It showed that the intervention (video version) resulted in significantly lower BMI (B = −0.25 with a Cohen’s d effect size of 0.10). The study also showed that both versions of the intervention (video and text) resulted in significantly lower average daily energy intake from energy-dense food products, compared to a control group (video, B = −175.58, Cohen’s d of 0.40; text, B = −163.05, Cohen’s d of 0.36), with no interaction effects of educational level on any of the outcome variables [20]. Yet, intervention effects of the 12-month follow-up are not yet investigated. More research is needed regarding its long-term effects, as evidence for the effectiveness of computer-tailored interventions regarding obesity and overweight are found only in the short term. It is thus of relevance to investigate the longitudinal effects of the computer-tailored intervention and potential differences in effects between the video- and text-based versions. Although no interaction effects were found in the 6-month follow-up of educational level on BMI, energy intake, and physical activity, it is relevant to explore potential longitudinal effects of educational level on outcomes. 

The aim of this study is twofold. The first aim is to assess the 12-month effectiveness of a video- and text-based computer-tailored intervention on BMI, energy intake, and physical activity [16]. The second aim is to explore the role of educational level in intervention effects regarding BMI, energy intake, and physical activity. 

## 2. Materials and Methods

The study protocol has been approved by the Ethical Committee of Open University Heerlen, and registered in the Dutch Trial Register (NTR3501). 

### 2.1. Computer-Tailored Intervention for Obesity Prevention

The computer-tailored intervention for obesity prevention was web-based and systematically developed in line with the Intervention Mapping protocol [21]. The intervention consists of two versions, video-based and text-based, and is aimed to prevent weight gain or achieve modest weight loss by enhancing energy intake, physical activity, or both. The video version delivered most of the educational content via videos with actors providing the tailored information, which was identical to the information that was delivered via text in the text version. The intervention tailors specific health messages to the participant’s answers to online questions regarding energy intake, physical activity level, and socio-cognitive constructs (e.g., intention and self-efficacy). Derived from self-regulation theories [22,23] and the I-Change Model [24], the intervention includes several behavior-change techniques such as goal setting, action and coping planning, evaluation of progress toward goal achievement, consciousness raising, and tailored feedback on behavior and cognitions. There were six weekly sessions of approximately 15 min: (1) session one informed participants about the different intervention sessions, provided feedback regarding their weight, behavior, and socio-cognitive beliefs, and asked participants to set goals; (2) session two provided participants with feedback on the chosen behavior and asked participants to make if-then plans specifying when, where, and how they would take specific actions to realize the behavior change; (3) session three provided participants the option to make coping plans and provided them tailored feedback regarding their change progress in order to realize and maintain behavior changes; and (4) sessions four to six were similar to session three with slight changes, introducing new elements to the intervention (e.g., role models narrating about their own change process and how they dealt with difficult situations). A more detailed description about the intervention development and its content is detailed in another study [16].

### 2.2. Design and Participants

A three-armed randomized controlled trial in The Netherlands was conducted, in which Dutch adults were allocated to the video-based condition, the text-based condition, or the control condition. Primary outcomes were self-reported body mass index (BMI), physical activity, energy intake and appreciation of the intervention. Measurements took place at baseline and 6 and 12 months after baseline. Inclusion criteria were: being minimal 18 years of age, being employed (because of initial recruitment procedure), having sufficient command of the Dutch language, and having a BMI higher than 18.5 kg/m^2^. As detailed in the previous study, 2000 participants at baseline were estimated to be needed in order to be able to detect a medium-sized effect [16,20]. In order to understand potential BMI differences in intervention effects, we included participants with a BMI higher than 30 kg/m^2^.

### 2.3. Procedure

From September 2012 until February 2013, participants were recruited during medical screenings via occupational health centers, companies, and advertisements in national and local newspapers. The recruitment information included a hyperlink to the intervention website. Participants were then asked to register and give informed consent online. Participants were randomly assigned in a computer-determined sequence to one of the three groups. Participants were then asked to fill in the baseline questionnaire (T0). In the intervention conditions, participants were given access to the intervention two weeks after completion of the baseline questionnaire. The interventions were available for a maximum period of three months. Follow-up mail was sent out 6 months after baseline (T1), and 12 months after baseline (T2). In order to minimize attrition, participants had a chance to win one €100 reward after completion of all questionnaires [25]. 

### 2.4. Measurements

All demographic characteristics and socio-cognitive variables were assessed at T0, while all outcome variables (i.e., BMI, energy intake, and physical activity) were assessed using online self-reports at baseline (T0), 6-month follow-up (T1), and 12-month follow-up (T2). The demographic characteristics, socio-cognitive variables, and outcome variables are briefly described below; see previous (6 months effectiveness) study for a more detailed description [20]. 

Participants were asked to report on their gender (i.e., male/female), age (in years), and educational level (i.e., highest level of education completed, which was categorized into low, medium, and high).

The socio-cognitive variables were self-efficacy, intention, and self-regulation skills. To compute the scales of each construct, the mean score was calculated from the corresponding items on a 5-point Likert scale. Self-efficacy was separately assessed for physical activity and energy intake using four items per behavior. Intention was also separately assessed, with one item per behavioral outcome, by asking participants if they intended to improve their diet or physical activity level within the next six months. Self-regulation skills are skills that enhance translating intentions into behavior change, consisting of goal setting (i.e., three items assessing whether participants set a goals in advance), action planning (i.e., three items assessing whether participants had a clear plan when, where, and how they wanted to improve their diet and physical activity), monitoring (i.e., four items assessing participants to which degree they monitored their weight and behavior on a regular basis), and coping planning (i.e., two items assessing to which degree they were able to identify hindering situations in advance and thought that they were able to deal with these situations, for both energy intake as well as physical activity).

The outcome measures were BMI, energy intake, and physical activity. BMI was estimated by assessing the participant’s body weight in kilograms (in the morning without clothes) and height in meters. Energy intake was assessed using a validated food frequency questionnaire consisting of 66 items [26], assessing intake levels of mainly energy-dense products, type of product (when applicable), and quantity. The average daily intake was calculated for each food product (where we assessed the number of days per week and servings per day), which was then combined with the energy value of the product in order to calculate a score for the average daily intake of calories from these products. Physical activity was assessed with the use of a validated questionnaire, the Short Questionnaire to Assess Health-Enhancing Physical Activity (SQUASH) [27]. For several categories (e.g., commuting activities and leisure time activities), the SQUASH assesses the participant’s level of physical activity (i.e., days per week engaged, average time spent, and intensity of the activity (light, moderate, or vigorous)). To estimate the average daily minutes of moderate-to-vigorous intensity physical activity, a total score was then calculated from these items.

### 2.5. Statistical Analysis

For all analyses, the open-source statistical software programming language R [28] was used. For post hoc comparisons, dummy variables were calculated for study condition (with the control group serving as the reference category) and educational level (with the lowest category serving as the reference category). The reference category for the dichotomous gender variable was male. Furthermore, a drop-out analysis was done, using a logistic regression (e.g., 1 = drop-out, 0 = no drop-out). Variables that were significantly associated with drop-out (e.g., goal setting) were included as covariates in all models (see Section 3). 

#### 2.5.1. Handling of Missing Values

For the estimation of the models, we used mixed-effects modelling where estimation is based on full information maximum likelihood (FIML). This allows keeping cases in the data with missing values on the dependent variable (e.g., respondents with a missing value on BMI on one or multiple time points). While it has been shown that FIML yields similar estimates as an analysis where estimates are pooled after multiple imputation, ideally both FIML and multiple imputation should be used [29]. Therefore, before estimating fixed and random effects in the mixed-effects model, we applied multiple imputation. The models were subsequently estimated on each data set using the same procedure that would have been used if the data had been complete, and the resulting multiple set of parameter estimates, standard errors, and *p* values were combined into a single set of results by pooling them. For the multivariate imputation (MI) procedure, we used the multivariate imputation by chained equation (MICE) package [30] that generates multivariate imputations using chained equations and a total of 50 imputed data sets. For the imputation model, all variables that were deemed relevant for the analysis as well as the missing mechanism were included. Due to computational issues, post hoc comparisons were based on the incomplete data set, but inspection of the 50 imputed data sets showed that the variance between imputed values was very low, meaning that similar estimates would have been obtained if multiple imputation would have been applied. 

#### 2.5.2. Mixed-Effects Modelling

In a linear mixed-effects model, responses from a respondent are thought to be the linear combination of so-called fixed and random effects. Fixed effects represent effects that affect only the population mean, and random effects (e.g., subject effects) are associated with a sampling procedure and therefore contribute to the covariance structure of the observed data. By doing so, time can be treated as a continuous variable instead of considering multiple linear regressions, and intra-individual variations can be modelled [31]. The analysis was conducted using the linear and non-linear mixed effects (nlme) R package [32]. 

#### 2.5.3. Model Selection

Three different models were estimated: one including BMI as dependent variable, one including energy intake as dependent variable, and one including physical activity as dependent variable. To find the model for our data with the lowest number of parameters (resulting in the largest degrees of freedom) along with the best fit, we tested various models and compared the Information Criterion (AIC) of all models. In this instance, we could reduce the number of parameters needed to be estimated and keep the model as simple as possible. This was done separately for all three models. 

First of all, to choose the best fitting covariance structure, we compared the AIC of the same simple model (i.e., including only the fixed main and the subject’s random intercept), assuming the following different covariance structures: (1) Autoregressive (AR) heterogeneity, (2) AR homogeneity, and (3) Compound symmetry. While the two AR models assume that measures at different time points are correlated in a certain way (i.e., the correlation decreases as time points get further apart), the third model does not assume any correlation between time points. The third model allows for heterogeneous variances at different time points, while the other models assume constant variance (i.e., the same variance at all time points). While earlier research suggests that the first (AR heterogeneity) is the most reasonable choice for (psychological) longitudinal data [33], a model assuming this kind of covariance structure is computationally extensive and needs a lot of parameters, which results in a loss of degrees of freedom. 

Second, the hypothesized model corresponding to our research question was compared to more simple models. In the hypothesized model (i.e., the model that corresponds to our research hypotheses), main effects were included for time, age, study condition (e.g., text, video, or control group), BMI category (i.e., BMI of < 18.5, BMI between 18.5 and 25, BMI between 25 and 30, or BMI higher than or equal to 30), gender, goal setting, and educational level (e.g., low, middle, or high). Furthermore, an interaction (moderator) between study condition and time, study condition and age, study condition and gender, study condition and BMI category, and study condition and educational level was added. The alternative models consisted of (1) a model that included only the main effects (alternative model 1), (2) all main effects and an interaction between study condition and time (alternative model 2), (3) all main effects and an interaction between study condition and age (alternative model 3), (4) all main effects and an interaction between study condition and gender (alternative model 4), (5) all main effects and an interaction between study condition and educational level (alternative model 5), and (6) all main effects and an interaction between study condition and BMI category (alternative model 6). In all models, a fixed intercept and a random intercept (varying per respondent) was included. The final model was also compared to a model that did not include a random intercept, a model that also included a random slope, and a model that included both (i.e., random intercept and random slope). After having chosen the best model for our data, post hoc analyses (i.e., pairwise comparisons) were performed for all significant categorical predictors in the form of contrasts among estimated least-squares means, using the R package lsmeans [34]. The Tukey method was applied to correct for multiple testing. The R syntax used for all analyses can be obtained on request from the second author of this manuscript. 

## 3. Results

### 3.1. Study Sample and Attrition Analysis

**Figure 1:**
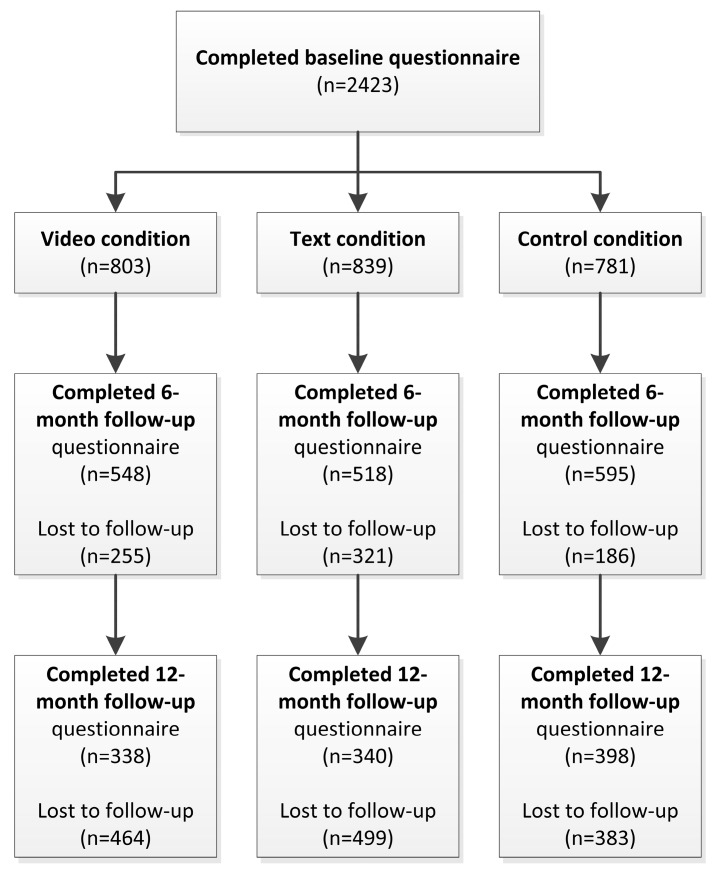
Flowchart of assessments.

Figure 1 details the number of participants for each assessment point. In total, 2423 participants completed the baseline questionnaire before they were randomized to one of the three study conditions. Data from 1076 participants (44.4%) was collected at the 12-month follow-up. There were no participants with a BMI lower than 18.5, 376 (16% of all known BMI values) participants with a BMI between 18.5 and 25, 1043 (44% of all known BMI values) with a BMI between 25 and 30, and 930 (39% of all known BMI values) with a BMI of 30 or higher. 

In the attrition analyses, several significant predictors of drop-out were identified at the 12-month follow-up. Compared to participants in the control condition, participants in the video (OR 0.64, 95% CI 0.51–0.79, *p* = 0.00) and text conditions (OR 0.57, 95% CI 0.46–0.71, *p* = 0.01) were significantly more likely to drop out. Compared to highly educated participants, participants with a low educational level were significantly more likely to drop out (OR 0.72, 95% CI 0.56–0.92, *p* = 0.01). With decreasing age, participants were more likely to drop out (OR 1.02, 95% CI 1.01–1.03, *p* = 0.00). In addition, higher attrition was found in participants who had higher levels of goal setting (OR 0.82, 95% CI 0.73–0.93, *p* = 0.00).

### 3.2. Descriptive Statistics

Table 1 details the baseline sample characteristics and the outcome estimates at baseline, 6-month follow-up, and 12-month follow-up. In addition, the number of missing data is provided.

### 3.3. Final Model

Based on a comparison of all AIC values, it was decided to keep the hypothesized full model as the preferred model for both energy intake and physical activity. For BMI, alternative model 6 was the preferred model, which contained main effects and an interaction between study condition and BMI category. Furthermore, for all three outcome variables, it was chosen to assume AR homogeneity for the covariance structure, as this model resulted in the lowest AIC value when compared to models with alternative covariance structures. A table of the fit statistics of the various models is available from the authors.

### 3.4. Intervention Effects on BMI and Physical Activity

The mixed effects models revealed, for both BMI (*p* = 0.44) and physical activity (*p* = 0.49) as dependent variable in the models, no significant main effects for study condition, which means that no differences in BMI or physical activity between the video-based, text-based, and control condition were found. Moreover, the interactions between study condition and time were also found to be not significant (*p* = 0.76), indicating that differences in BMI or physical activity between the three conditions did not significantly change over time. Results thus showed no significant intervention effects on BMI and physical activity after the 12-month follow-up.

Regarding BMI, significant effects were found for time (*p* = 0.00), educational level (*p* = 0.00), gender (*p* = 0.00), and goal setting (i.e., higher levels of goal setting corresponded to lower BMI) (*p* = 0.01). Post hoc analyses for the categorical variables—performed to assess differences in BMI between the levels of the categorical variables—showed that in comparison with the baseline, participants had lower BMI at the 6-month follow-up (least squares means (coefficient) difference (LSM-diff) = 0.67, (SE = 0.06), t(1339) = 10.92, *p* = 0.00) and at the 12-month follow-up (LSM-diff = 0.70, (SE = 0.08), t(1339) = −9.22, *p* = 0.00). No significant BMI differences were found between 6 and 12 months (*p* = 0.88). Next to that, lower educational levels corresponded to higher BMI (lower education vs. medium educational level, LSM-diff = 0.97 (SE = 0.29), t(2271) = 3.33, *p* = 0.00; lower education vs. higher educational level, LSM-diff = 3.04, (SE = 0.28), t(2271) = 10.83, *p* = 0.00; medium education vs. higher educational level, LSM-diff = 2.06, (SE = 0.23), t(2271) = 8.88, *p* = 0.00). Furthermore, post hoc analyses showed that male participants had higher BMI than female participants (LSM-diff = 0.66 (SE = 0.21), t(2271) = 3.11, *p* = 0.00). Effect sizes of significant differences measured by Cohen’s D were 0.31 (95% CI 0.26–0.37) (baseline vs. 6-month follow-up), 0.26 (95% CI 0.21–0.32) (baseline vs. 12-month follow-up), 0.11 (95% CI 0.04–0.17) (lower education vs. medium education level), 0.34 (95% CI 0.28–0.40) (lower education vs. higher education level), 0.24 (95% CI 0.18–0.29) (medium education vs. higher education level), and 0.07 (95% CI 0.03–0.12) (men vs. women). 

Regarding physical activity, significant effects were found for time (*p* = 0.03), educational level (*p* = 0.02), age (i.e., higher age corresponds to higher levels of physical activity) (*p* = 0.03), and goal setting (i.e., higher levels of goal setting correspond to higher levels of physical activity) (*p* = 0.00). Post hoc analyses for the categorical variables showed that participants at baseline had lower levels of physical activity than at the 6-month follow-up (LSM-diff = −34.51, (SE = 3.32), t(1342) = −4.36, *p* = 0.00) and at the 12-month follow-up (LSM-diff = −12.97, (SE = 2.98), t(1342) = −10.38, *p* = 0.00). Participants in the 12-month follow-up did have lower levels of physical activity than those in the 6-month follow-up (LSM-diff = 21.54, (SE = 3.91), t(1342) = 5.51, *p* = 0.00). Moreover, lower educational levels correspond to higher levels of physical activity (lower education vs. medium educational level, LSM-diff = 12.71, (SE = 4.98), t(2306) = 2.55, *p* = 0.03; lower education vs. higher educational level, LSM-diff = 30.08, (SE = 4.79), t(2306) = 6.28, *p* = 0.00; medium education vs. higher educational level, LSM-diff = 17.37, (SE = 3.92), t(2306) = 4.43, *p* = 0.00). Effect sizes of significant differences measured by Cohen’s D were 0.30 (95% CI 0.24–0.35) (baseline vs. 6-month follow-up), 0.13 (95% CI 0.07–0.18) (baseline vs. 12-month follow-up), 0.16 (95% CI 0.10–0.21) (6-month follow-up vs. 12-month follow-up, 0.08 (0.02–0.15 95% CI) (lower education vs. medium education level), 0.19 (95% CI 0.14–0.26) (lower education vs. higher education level), and 0.12 (95% CI 0.06–0.17) (medium education vs. higher education level). 

### 3.5. Intervention Effects on Energy Intake

Regarding energy intake as dependent variable, main effects for study condition was shown not to be significant (*p* = 0.06), but a significant interaction was identified between study condition and time (*p* = 0.02). Hence, post hoc analyses were relevant to understand potential time differences in intervention effects. Table 2 depicts the results of the main and interaction effects for energy intake as dependent variable, while Table 3 details the post hoc analysis for study condition and time regarding energy intake. The post hoc analyses showed that participants in both intervention conditions (i.e., video and text) had significantly lower calorie intake compared to the control condition. The effect remained significant for the video intervention in the 12-month follow-up, whereas the effects from the text intervention were not significant in the 12-month follow-up. Effect sizes of the significant differences as measured by Cohen’s D were 0.20 (95% CI 0.10–0.30) (video vs. control at 6-month follow-up), 0.24 (95% CI 0.15–0.34) (text vs. control at 6-month follow-up), and 0.12 (95% CI 0.02–0.15) (video vs. control at 12-month follow-up).

Furthermore, main effects were also found for time, gender, and goal setting, and an interaction effect was found for study condition and age. Post hoc analyses were conducted for time, gender, and the interaction between study condition and age; goal setting was not a categorical variable and thus not included in the post hoc analyses. At baseline, participants had the highest levels of energy intake (baseline vs. 6-month follow-up, LSM-diff = 269.78 (SE = 15.64), t(1369) = 17.25, *p* = 0.00; baseline vs. 12-month follow-up, LSM-diff = 220.05, (SE = 15.24), t(1369) = 14.44, *p* = 0.00). However, in the 6-month follow-up, participants had lower levels of energy intake compared to the 12-month follow-up (LSM-diff = 49.72 (SE = 18.18), t(1369) = −2.74, *p* = 0.02). Post hoc analyses also revealed that male participants had higher levels of calorie intake compared to female participants (LSM-diff = 352.26 (SE = 20.78), t(1369) = 16.95, *p* = 0.00). Moreover, the interaction of study condition and age showed that—when age is averaged (i.e., 49.07)—participants in the text condition had lower levels of calorie intake than participants in the control condition (LSM-diff = 78.60 (SE = 29.10), t(1369) = −2.701, *p* = 0.01). Effect sizes of significant differences measured by Cohen’s D were 0.50 (95% CI 0.44–0.55) (baseline vs. 6-month follow-up), 0.41 (95% CI 0.36–0.47) (baseline vs. 12-month follow-up, 0.08 (95% CI 0.02–0.12) (6-month follow-up vs. 12-month follow-up), 0.40 (95% CI 0.36–0.45) (men vs. women), and 0.31 (95% CI 0.08–0.53) (text vs. control) for respondents with and without an average age of 49.07.

## 4. Discussion

### 4.1. Main Findings

This study assessed the 12-month effects of a video- and text-based computer-tailored intervention while also exploring the role of educational level on the intervention effects regarding BMI, energy intake, and physical activity. Regarding BMI and physical activity, the intervention had no significant effects after one year. However, for energy intake, the interaction between study condition and time was found to be significant. 

After one year, the video intervention led to significantly better energy intake (i.e., lower amounts of consumed calories) compared to the control group. The effect of the video intervention did not vary across educational roles and BMI, indicating that this intervention may be valuable to make small changes in energy intake among participants with lower as well as higher educational levels, and lower as well as higher BMI categories. In line with the previous 6-month study [20], the video version of the intervention was more effective after 12 months in enhancing energy intake (i.e., lowering calorie intake) than the text version. This was illustrated by a diminished effect of the text version after one year, whereas the video version still had effects on energy intake after one year. More research may shed light on how to prevent a reduction in effects and intervention use over time, for instance by exploring the potential of booster sessions which may be an effective tool for maintaining or increasing intervention effects [35]. Our results are also consistent with previous studies on web-based computer-tailored interventions comparing video and text versions [36,37]. One explanation may be the better appreciation of the video version compared to the text version [20], as according to the Elaboration Likelihood Model it is more likely for a person to process information via the central route when information is perceived as interesting and attractive, resulting into more long-lasting persuasive effects. 

Intervention effects on BMI could not be maintained, which is not surprising as the effect was already borderline significant at the 6-month follow-up in the previous study [20]. Although the video intervention had long-term effects on energy intake, the effect is less strong compared to the 6-month follow-up, as reflected in post hoc analyses. This may partly explain the diminished (small) effect on BMI after one year. In addition, literature indicates that physical activity is an important factor in maintaining weight loss, next to dietary intake [38]. The lack of intervention effects found on physical activity may thus also explain the diminished effect on BMI after one year. Next to that, the lack of intervention effects on physical activity is not surprising, as the intervention did not yield effects on physical activity in the 6-month follow-up study [20]. One reason for the absence of this effect could be that the measurement of physical activity by the SQUASH list (average daily minutes of moderate-to-vigorous intensity activity) resulted in unrealistically high scores. Consequently, the intervention did not advise many participants to increase their physical activity, which calls for more reliable measures of physical activity and adjusting the algorithms of the tailored intervention [39]. 

The long-term effectiveness of the video version indicates important public health potential to improve energy intake on a large scale. Yet, the significant effect had a small effect size after one year. However, the translation of effect size estimates to the assessment of practical importance is not straightforward. Many considerations of the context should be factored into assessments of practical importance [40], and within the context of public health, even small effects in estimates are relevant. Despite the small effect size, the intervention may have a large public health impact due to the prevention of many health problems and potential improved quality of life [41]. Yet, due to the increasing number of lifestyle interventions and the limited resources available, policy makers are forced to make choices regarding reimbursements. Literature showed that behavior-change interventions of obesity prevention, including interventions focusing on energy intake (e.g., reducing calorie intake) are cost-effective and may even be cost-saving [42]. The video version of the intervention is now evidence-based and may be a good candidate for decision-making, as effectiveness is already shown and internet-based interventions have high opportunities to be cost-effective due to the low costs per person. Future research is needed to evaluate the cost-effectiveness of the web-based tailored intervention, and may even model the lifetime cost-effectiveness. Furthermore, no differences were found between educational level on the intervention effects, which is consistent with literature showing that no significant differences were found between groups with different levels of education regarding e-Health intervention use as recommended [14]. Findings of this study thus suggest the potential to implement the video intervention for Dutch adults of all levels of education. 

### 4.2. Strengths and Limitations

One strength of this study is the examination of the long-term effectiveness of a video-based online computer-tailored intervention, which was developed using the Intervention Mapping protocol [21]. Another strength is the analysis via linear mixed-effects models [31], in which fixed and random effects are combined in the model, which thus contributes to the covariance structure of the observed data (meaning that intra-individual variations between the assessment points can be modelled [31]). This study also has several limitations. As pointed out in the 6-month follow-up study, one limitation may be that all outcome measures relied on self-reports rather than on objective measures [43]. The problem with self-reports seemed to be reflected by physical activity measures [44], with unrealistically high physical activity scores which resulted in a lack of tailored feedback regarding their physical activity. More research is needed to identify the best practice to overcome this overestimation, such as adjusting tailoring algorithms or the usage of objective measures. Another limitation is the potential lack of generalizability of the study sample to Dutch adults (or beyond). The drop-out analysis showed some significant predictors, including educational level and study condition. Participants with lower to medium educational levels, and participants in the intervention conditions, were more likely to drop out. This is not surprising as participants in the intervention conditions had a higher burden to provide input compared to the participants in the control condition, and that previous studies regarding web-based computer-tailored interventions showed higher attrition rates among participants with a lower or middle educational level, compared with highly educated participants [45]. Yet, this means a potential sample bias, especially regarding participants with a lower or middle educational level. Findings regarding educational differences should therefore be interpreted with caution. 

## 5. Conclusions

The video version of the intervention was more effective than the text version of the web-based computer-tailored intervention and control group regarding energy intake after one year. Intervention effects did not to differ between educational levels and BMI categories, suggesting the broad implementation potential. Due to the low costs per person for internet-based interventions, the intervention may be highly cost-effective and even cost-saving. More research regarding the cost-effectiveness of the intervention is needed to create a pulse for the reimbursement and implementation of such internet-based obesity prevention interventions. Additional research is needed to prevent a reduction in effects and intervention use over time, for instance by assessing the potential of booster sessions. Future research is also needed to shed light how to realize changes in BMI and physical activity.

## Figures and Tables

**Table 1:** Baseline sample characteristics and outcome estimates at baseline and follow-ups.

Variable	Video (*n* = 803)	Missing	Text (*n* = 839)	Missing	Control (*n* = 781)	Missing
Gender (male), *n* (%)	338 (42.1%)	0	360 (42.9%)	0	316 (40.5%)	0
Educational level, *n* (%)						
Low	166 (20.7%)	0	167 (19.9%)	0	158 (20.2%)	0
Middle	257 (32.0%)	0	290 (34.6%)	0	296 (37.9%)	0
High	380 (47.3%)	0	382 (45.5%)	0	327 (41.9%)	0
Age, M (SE)	48.27 (11.57)	0	47.95 (11.36)	1	48.63 (11.31)	4
**Baseline**						
BMI, M (SE)	29.77 (5.19)	17	29.74 (5.12)	16	29.32 (4.95)	41
Energy intake, M (SE)	1384.11 (557.70)	9	1351.46 (538.28)	11	1320.59 (585.82)	22
Physical activity, M (SE)	75.65 (81.96)	0	76.23 (83.39)	0	81.23 (91.22)	0
**Six-month follow-up**						
BMI, M (SE)	28.79 (5.09)	262	28.70 (4.91)	333	28.58 (4.75)	205
Energy intake, M (SE)	1014.88 (445.03)	621	990.25 (487.60)	654	1189.77 (548.16)	431
Physical activity, M (SE)	109.15 (101.32)	621	115.32 (101.71)	657	114.99 (116.51)	439
**Twelve-month follow-up**						
BMI, M (SE)	28.47 (5.17)	466	28.22 (4.60)	504	28.72 (5.09)	388
Energy intake, M (SE)	1073.94 (414.50)	598	1101.00 (424.41)	622	1158.61 (435.78)	488
Physical activity, M (SE)	91.89 (88.36)	614	89.69 (84.61)	632	102.24 (103.59)	505

BMI: body mass index; SE: standard error; M: mean.

**Table 2:** Multilevel analysis with energy intake as dependent variable.

Variable	B	SE	95% CI (Lower)	95% CI (Higher)	*p*-Value
Intercept	2103.45	165.50	1777.59	2429.31	0.00
Study condition	136.18	70.95	−3.33	275.68	0.06
Time	−29.05	3.75	−36.43	−21.67	0.00
Educational level	−49.46	27.42	−103.38	4.45	0.07
Gender	−271.71	44.82	−359.93	−183.49	0.00
Age	−1.96	1.90	−5.70	1.77	0.30
Goal setting	−33.46	10.49	−54.19	−12.73	0.00
BMI category	79.03	76.97	−72.14	230.20	0.31
Condition * time	3.88	1.64	0.66	7.10	0.02
Condition * educational level	−8.63	12.19	−32.58	15.32	0.48
Condition * gender	−25.01	19.84	−64.01	14.00	0.21
Condition * age	−1.82	0.83	−3.45	−0.19	0.03
Condition * BMI category	−68.37	74.73	−215.12	78.39	0.36

B = Least squares means; SE = standard error; *: interaction.

**Table 3:** Post hoc analysis for study condition and time with energy intake as dependent variable.

Contrast	LSM-diff	SE	*p*-Value
Baseline			
Video vs. Control	39.69	44.49	0.57
Text vs. Control	19.72	27.70	0.69
6-month follow-up			
Video vs. Control	−205.40	52.38	0.00
Text vs. Control	−198.44	40.54	0.00
12-month follow-up			
Video vs. Control	−128.14	53.17	0.03
Text vs. Control	−57.10	40.18	0.27

LSM-diff = Least squares means difference; SE = standard error.
